# miR-218 Inhibits Erythroid Differentiation and Alters Iron Metabolism by Targeting *ALAS2* in K562 Cells

**DOI:** 10.3390/ijms161226088

**Published:** 2015-11-26

**Authors:** Yanming Li, Shuge Liu, Hongying Sun, Yadong Yang, Heyuan Qi, Nan Ding, Jiawen Zheng, Xunong Dong, Hongzhu Qu, Zhaojun Zhang, Xiangdong Fang

**Affiliations:** 1 CAS Key Laboratory of Genome Sciences and Information, Beijing Institute of Genomics, Chinese Academy of Sciences, Beijing 100101, China; liym@big.ac.cn (Y.L.); 18101354208@163.com (S.L.); hongying.sun@rochester.edu (H.S.); yangyd@big.ac.cn (Y.Y.); qihy@im.ac.cn (H.Q.); dingnan@big.ac.cn (N.D.); zhengjw@big.ac.cn (J.Z.); dongxn@big.ac.cn (X.D.); quhongzhu@big.ac.cn (H.Q.); zhangzhaojun@big.ac.cn (Z.Z.); 2 College of Life Sciences, University of Chinese Academy of Sciences, Beijing 100049, China

**Keywords:** miR-218, *ALAS2*, erythroid differentiation, iron metabolism

## Abstract

microRNAs (miRNAs) are involved in a variety of biological processes. The regulatory function and potential role of miRNAs targeting the mRNA of the 5′-aminolevulinate synthase 2 (*ALAS2*) in erythropoiesis were investigated in order to identify miRNAs which play a role in erythroid iron metabolism and differentiation. Firstly, the role of *ALAS2* in erythroid differentiation and iron metabolism in human erythroid leukemia cells (K562) was confirmed by *ALAS2* knockdown. Through a series of screening strategies and experimental validations, it was identified that hsa-miR-218 (miR-218) targets and represses the expression of *ALAS2* by binding to the 3′-untranslated region (UTR). Overexpression of miR-218 repressed erythroid differentiation and altered iron metabolism in K562 cells similar to that seen in the *ALAS2* knockdown in K562 cells. In addition to iron metabolism and erythroid differentiation, miR-218 was found to be responsible for a reduction in K562 cell growth. Taken together, our results show that miR-218 inhibits erythroid differentiation and alters iron metabolism by targeting *ALAS2* in K562 cells.

## 1. Introduction

Red blood cells are critical to human survival due to their function in delivering oxygen to all cells and organs of the body. A functional red blood cell depends on hemoglobin, which consist of four globin units and four iron-containing hemes. Successful erythropoiesis, therefore, requires the coordinated regulation of globin gene expression, iron metabolism, and heme synthesis.

miRNAs are a family of small non-coding RNAs which regulate gene expression by binding the 3′UTR of mRNAs resulting either in inhibition of gene expression or mRNA degradation. Previous studies have partially revealed the role of miRNAs in erythropoiesis. In stem cells, miR-24 targets and inhibits type I activin receptor (*ALK4*), repressing the transforming growth factor-β (TGF-β)/Smad pathway which is involved in the expression of β-globin [1]. miR-126 and miR-15a have been shown to repress *c-Myb*, a transcription factor which promotes the expression of critical erythroid specific transcription factors like Kruppel-like factor 1 (*KLF1*) and GATA binding protein 1 (*GATA-1*), proteins required for erythropoiesis, like type-3 transmembrane receptor for mast cell growth factor, *KIT*, and LIM domain only 2 (*LMO2*) [2,3]*.* MiR-221/222 also targets *KIT*, an essential gene in erythroid cell proliferation and differentiation, and represses its expression [4]. In committed erythroid cells, *GATA-1* promotes expression of miR-451, which inhibits *GATA-2* [5,6]. During the late stage of erythroid differentiation, the inhibition of RIO kinase 3 (*Riok3*) and *Mix1* (a c-Myc antagonist [7]) by miR-191 is relaxed in order to allow for enucleation [8].

Cellular iron homeostasis is mainly controlled by an Iron Regulatory Protein (IRP)/Iron-Responsive Element (IRE) regulatory network [9,10,11]. This network comprises two RNA binding proteins (IRP1 and IRP2) and cis-regulatory RNA elements (IRE) that are present in mRNAs encoding for proteins involved in iron homeostasis [9,12]. When IRP binds at the 5′UTR of iron storage protein genes (e.g., ferritin, heavy polypeptide 1, *FTH1* and ferritin, light polypeptide, *FTL*) [13], the heme synthesis enzyme gene (5′-aminolevulinate synthase 2, *ALAS2*) [10], and the iron export gene (ferroportin*,* *SLC40A1*) [14], the translation initiation of these genes will be inhibited. When IRP binds at the 3′UTR of iron-uptake protein genes (e.g., transferrin receptor 1, *TFR1*, and divalent metal transporter 1, *DMT1*), the stabilization of theses mRNAs is enhanced [15].

To date, little is known about the regulation of iron metabolism by miRNAs in erythroid cells. Only one study has been published which indicates that miR-let-7d participates in the finely-tuned regulation of iron metabolism by targeting the *DMT1-IRE* isoform in erythroid cells [16].

In this present study, we investigated the role miRNA(s) play in erythroid differentiation and related erythroid iron metabolism. The first enzyme involved in the synthesis of heme is 5′-aminolevulinate synthase 2, encoded by the *ALAS2* gene, and, as such, is critical for heme synthesis and erythrocyte function. *ALAS2* is also responsible for iron metabolism through IRP binding the IRE in the 5′UTR of *ALAS2* mRNA [10]. It was assumed that miRNA(s) regulating *ALAS2* in erythroid cells could play a role in erythroid differentiation and related erythroid iron metabolism. With this in mind we screened candidate miRNAs from previously generated RNA-seq and miRNA-seq data from undifferentiated human embryonic stem cells (HESC), embryonic stem cells-derived erythroid cells (ESER), fetal liver-derived erythroid cells (FLER), and adult mobilized peripheral blood CD34+ cells-derived erythroid cells (PBER) [17]. Through a series of screening and functional studies, we found that miR-218 inhibited erythroid differentiation and altered iron metabolism by targeting *ALAS2* in K562 cells.

## 2. Results and Discussion

### 2.1. ALAS2 Knockdown Repressed Erythroid Gene Expression and Altered Iron Metabolism-Related Genes Expression in K562 Cells

**Figure 1:**
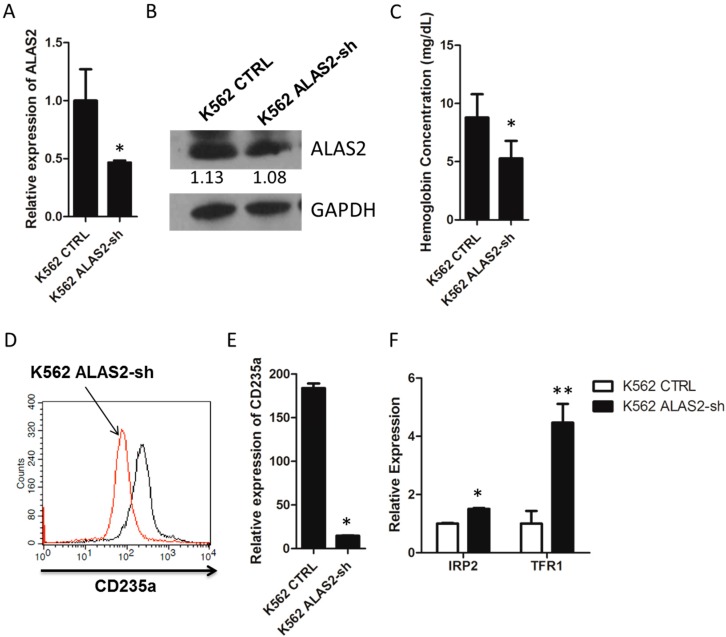
(**A**) Relative mRNA expression of *ALAS2* in K562 control and K562 ALAS2-sh cells using quantitative real-time PCR; (**B**) Representative Western blots showing ALAS2 expression in K562 control and K562 ALAS2-sh cells. Quantification normalized to GAPDH by densitometry using ImageJ was provided; (**C**) Hemoglobin concentration of K562 control and K562 ALAS2-sh cells. Cell surface expression of CD235a (**D**) and mRNA levels (**E**) of K562 control (black line in **D**) and K562 ALAS2-sh using FACS and quantitative real-time PCR, respectively; (**F**) Relative mRNA expression of *IRP2* and *TFR1* in K562 control and K562 ALAS2-sh cells using quantitative real-time PCR. All relative mRNA expression using quantitative real-time PCR was normalized to glyceraldehyde-3-phosphate dehydrogenase (*GAPDH*) expression. Asterisks indicate that differences between samples were statistically significant according to an independent-sample *t*-test, * *p* < 0.05, ** *p* < 0.01.

The human erythroid leukemia cell line K562 has been widely employed in erythroid differentiation studies for its response to hemin and compounds [18,19,20,21]. A siRNA specific to *ALAS2* was designed and cloned into pRNAT-U6.1/Neo as described in experimental section. K562 cells were transfected with pRNAT-U6.1/Neo or pRNAT-ALAS2-sh to generate a control (K562 CTRL) and knockdown cell line (K562 ALAS2-sh). Quantitative real-time PCR and Western blot were performed to confirm the knockdown of *ALAS2* (Figure 1A,B). Hemoglobin concentration was then measured in the K562 ALAS2-sh cells and was shown to be significantly lower than the control cells (Figure 1C). In addition, the erythroid marker CD235a was measured by FACS, indicating that the expression of CD235a on the K562 ALAS2-sh cell surface was significantly down-regulated compared to the control cells (Figure 1D). Quantitative real-time PCR also confirmed the decreased expression of CD235a in K562 ALAS2-sh cells (Figure 1E). With regard to iron metabolism, the effect of the *ALAS2* knockdown on the expression of *IRP2* and *TFR1* was also investigated*.* Results indicated that *IRP2* and *TFR1* expression were significantly up-regulated in the K562 ALAS2-sh cells (Figure 1F).

The role of *ALAS2* in erythroid differentiation and iron metabolism has been widely considered. Several studies have reported that patients with X-linked sideroblastic anemia (XLSA) carry mutations in the *ALAS2* gene [22,23,24,25]. The mutations are thought to be responsible for the development of hypochromic anemia with ring sideroblasts, characterized by mitochondrial iron accumulation. On the other hand, studies in mice and definitive erythroblasts indicate that *ALAS2* null mutations resulted in iron accumulation in the cytoplasm but not in mitochondria [26,27]. These studies confirmed *ALAS2* play a role in erythroid differentiation and iron metabolism, but the regulation effects in different study models remain for further investigation. Our study indicated that *ALAS2* knockdown decreased hemoglobin concentration, and increased *IRP2* and *TFR1* expression, which implied altered iron metabolism in K562 cells.

### 2.2. Candidate miRNAs Screening

**Figure 2:**
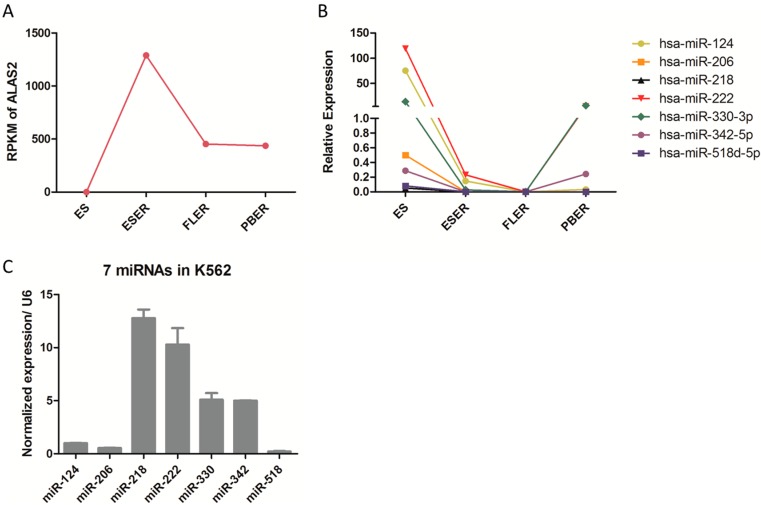
(**A**) RPKM (reads per kilobase of exon model per million mapped reads) value of *ALAS2* in cells at different erythroid differentiation and development stages. The data was obtained from our previous study; (**B**) The CF (cloning frequency) value of seven miRNAs in cells at different erythroid differentiation and development stages. The data was obtained from our unpublished study; (**C**) Relative expression of seven miRNAs in K562 cells examined by quantitative real-time PCR normalized to snRNA *U6*.

Having preliminarily confirmed the function of *ALAS2* in K562 cells, a search of databases for miRNA(s) which could regulate *ALAS2* was undertaken (Figure S1). Initially, 35 candidates were identified from four databases using prediction programs (Table 1). An additional seven candidates were picked from the miRNA-seq data generated in previous study that had opposite expression patterns to *ALAS2* (Figure 2A,B, Table S1). The seven miRNAs were hsa-miR-124, miR-206, miR-218, miR-222, miR-330, miR-342, and miR-518. In addition, the expression level of seven miRNAs in K562 cells were examined as basic information for further study in K562 cells (Figure 2C).

**Table 1:** miRNAs targeting *ALAS2* predicted by online miRNA databases.

miRNA	miRanda	microcosm	microrna.org	TargetScan
hsa-miR-1207-3p	-	-	-	√
hsa-miR-124	-	√	-	-
hsa-miR-1254	-	-	-	√
hsa-miR-1257	-	-	-	√
hsa-miR-140	√	-	-	-
hsa-miR-142-5p	√	√	-	-
hsa-miR-202	√	√	-	-
hsa-miR-206	√	-	-	-
hsa-miR-218	√	√	√	√
hsa-miR-222	√	-	-	-
hsa-miR-24	√	√	√	-
hsa-miR-298	-	√	-	√
hsa-miR-30a-3p	√	-	-	-
hsa-miR-30e-3p	√	-	-	-
hsa-miR-330	√	-	-	-
hsa-miR-330-3p	-	√	-	√
hsa-miR-342-5p	-	-	-	√
hsa-miR-378	-	√	-	-
hsa-miR-422a	-	√	-	-
hsa-miR-483-5p	-	√	-	-
hsa-miR-500	-	√	-	-
hsa-miR-506	√	√	-	-
hsa-miR-518d-5p	-	√	-	-
hsa-miR-520f	√	√	-	-
hsa-miR-526a	√	-	-	-
hsa-miR-565	√	√	-	-
hsa-miR-571	√	√	-	-
hsa-miR-608	-	-	-	√
hsa-miR-636	-	-	-	√
hsa-miR-661	-	√	-	-
hsa-miR-886-3p	-	√	-	-
hsa-miR-891a	-	√	-	-
hsa-miR-922	-	√	-	-
hsa-miR-92a-2*	-	√	-	√
hsa-miR-940		-	-	-

-: the miRNA is not predicted targeting *ALAS2* in the corresponding database. √: the miRNA is predicted targeting *ALAS2* in the corresponding database.

**Figure 3:**
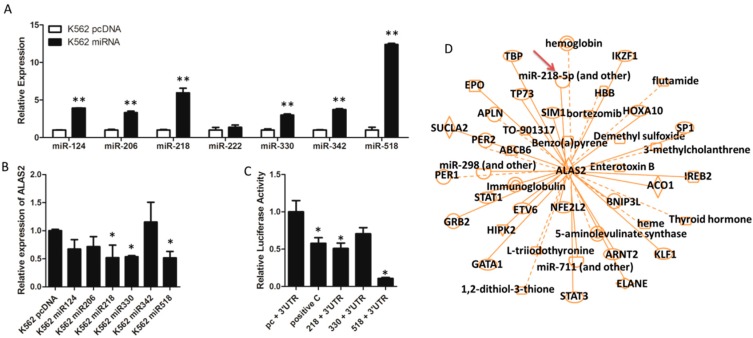
(**A**) Relative mature miRNA expression in K562 control cells and each cell line overexpressing one of the candidate miRNAs using quantitative real-time PCR normalized to snRNA *U6*; (**B**) Relative expression of *ALAS2* in control and each miRNA overexpression K562 cells using quantitative real-time PCR normalized to *GAPDH*; (**C**) K562 cells were transfected with candidate miRNA (or pcDNA3.0 as control) and the luciferase construct with the ALAS2-3′UTR. After 48 h, relative firefly/renilla luciferase activity in the cells was evaluated. Co-transfection of miR-124 and vimentin (*VIM*) 3′UTR was performed as a positive control [28] as shown in the second column. Asterisks indicate that differences between samples were statistically significant according to an independent-sample *t*-test, * *p* < 0.05, ** *p* < 0.01; (**D**) Functional network of *ALAS2* constructed by IPA. miR-218 was listed in the network and highlighted by the red arrow.

The seven pri-miRNA sequences were amplified and cloned into pcDNA3.0 to generate transient overexpression in the K562 cell line. Six of the cloned pri-miRNA sequences were successfully cloned and quantitative real-time PCR was performed to confirm their overexpression (Figure 3A). The diverse overexpression level (Figure 3A) may contribute from distinct expression pattern of seven miRNAs in K562 cells (Figure 2C). Further data revealed that *ALAS2* can be down-regulated directly by overexpression of miRNA-218, miRNA-518, and miRNA-330 (Figure 3B).

A dual-luciferase reporter assay was then performed to assess the binding of miRNA to the 3′UTR of *ALAS2*. The repressed relative luciferase activity indicated that miRNA-218 and miRNA-518 can bind the 3′UTR of *ALAS2* (Figure 3C). The functional network of *ALAS2* was further analyzed using the Ingenuity Pathways Analysis (IPA) system, which revealed that miR-218 and *ALAS2* are part of the same network (Figure 3D). For this reason miR-218 was chosen as a candidate for further study.

### 2.3. ALAS2 Is a Down-Regulated Target of miR-218

**Figure 4:**
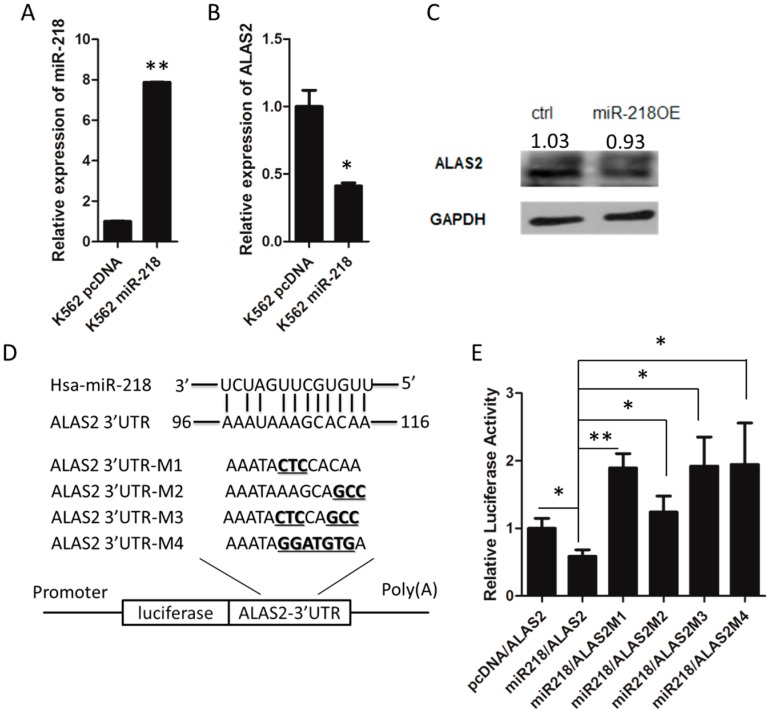
(**A**) The overexpression of miR-218 in K562 cells was confirmed using mature miRNA quantitative real-time PCR normalized to *U6*. The expression of *ALAS2* at the mRNA level (**B**) and at the protein level (**C**) in K562 control and K562 miR-218 cells was measured by quantitative real-time PCR and western blot, respectively. Western blot quantification normalized to GAPDH by densitometry using ImageJ was provided; (**D**) Predicted binding site for the seed sequence of miR-218 in the 3′UTR of *ALAS2* mRNA. Four mutated forms of the 3′UTR were used to generate luciferase reporter constructs as shown. The mutated nucleic acid was shown in bold and underlines; (**E**) miR-218 inhibits wild-type, but not the mutated *ALAS2* 3′UTR. Asterisks indicate that differences between samples were statistically significant according to an independent-sample *t*-test, * *p* < 0.05, ** *p* < 0.01.

A cell line (K562 miR-218) that stably overexpressed miR-218 was generated to further study the function of miR-218 in K562 cells (Figure 4A). Quantitative real-time PCR and Western blot analyses of K562 control and K562 miR-218 cells was performed and indicated that *ALAS2* was repressed both at the mRNA (Figure 4B) and protein level (Figure 4C) by miR-218. To further confirm miR-218 binding to the 3′UTR of *ALAS2*, four different mutations at the binding site were generated (Figure 4D). A dual-luciferase reporter assay indicated that the repression of luciferase activity was reversed when the wild type 3′UTR was replaced by a mutated 3′UTR (Figure 4E). A certain expression of miR-218 in K562 cells (Figure 2C) may be the mainly cause of higher luciferase activity of mutated groups than control group (pcDNA/ALAS2). These data revealed that *ALAS2* expression is down-regulated by miR-218 binding.

### 2.4. Iron Metabolism Is Regulated by miR-218 in K562 Cells

**Figure 5:**
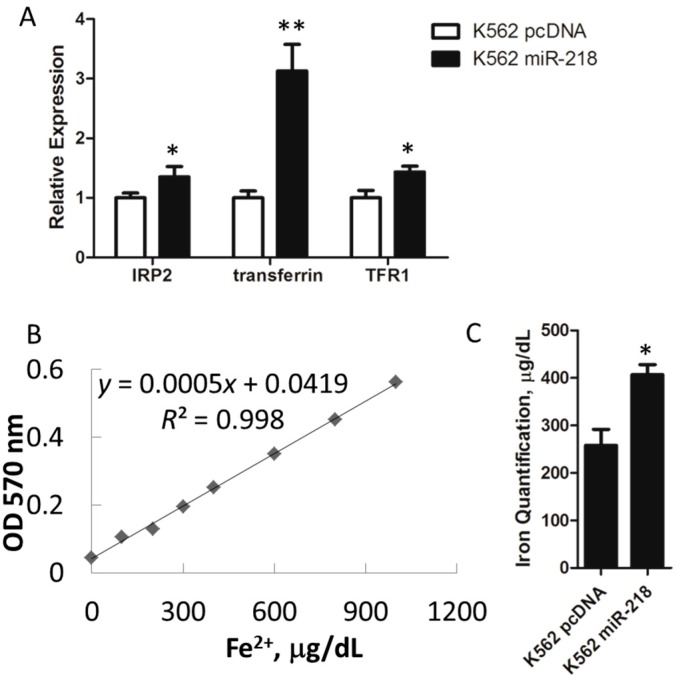
(**A**) Relative mRNA expression of iron metabolism related genes in K562 pcDNA and K562 miR-218 cells using quantitative real-time PCR; (**B**) Standard curve of iron concentration against relative absorption values at OD_570 nm_; (**C**) The iron concentration in the mitochondria of the K562 pcDNA and K562 miR-218 cells was calculated from the standard curve. All relative mRNA expression using quantitative real-time PCR was normalized to *GAPDH*. Asterisks indicate that differences between samples were statistically significant according to an independent-sample *t*-test, * *p* < 0.05, ** *p* < 0.01.

To assess the role of miR-218 in erythroid iron metabolism, the expression of several critical iron metabolism regulating genes was determined. Data revealed that *IRP2*, *transferrin* and *TFR1* were significantly up-regulated by miR-218 overexpression (OE) in K562 cells (Figure 5A). This expression level implied that the K562 miR-218 OE cells were in a situation of iron insufficiency.

Heme is synthesized from iron and protoporphyrin IX by ferrochelatase in the mitochondria. We further speculated whether the up-regulation of *IRP2*, *transferrin*, and *TFR1* was due to iron accumulation in mitochondria as a result of the down-regulation of *ALAS2*. Therefore, to validate this hypothesis, we isolated mitochondria from both K562 control and K562 miR-218 cells and calculated the iron concentration. Indeed, our results indicated that the amount of iron in the mitochondria from the K562 cells expressing miR-218 was higher than that in the control cells (Figure 5B,C).

### 2.5. Overexpression of miR-218 Inhibits Induced Erythroid Differentiation in K562 Cells

**Figure 6:**
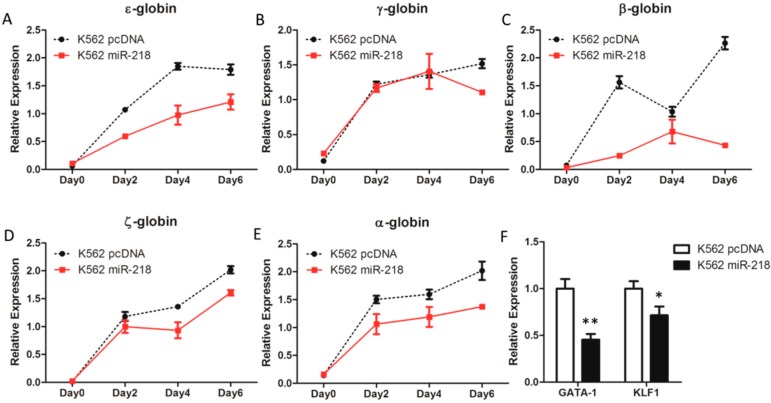
The relative mRNA expression of ε-globin (**A**), γ-globin (**B**), β-globin (**C**), ζ-globin, (**D**) and α-globin (**E**) gene during hemin-induced erythroid differentiation of K562 pcDNA and K562 miR-218 cells was determined using quantitative real-time PCR; (**F**) The expression of *GATA-1* and *KLF1* in K562 pcDNA and K562 miR-218 cells were also identified by quantitative real-time PCR. All relative mRNA expression using quantitative real-time PCR was normalized to *GAPDH* and β-*actin*. Asterisks indicate that differences between samples were statistically significant according to an independent-sample *t*-test, * *p* < 0.05, ** *p* < 0.01.

Hemin was used to induce K562 cell erythroid differentiation. Cells at different time points after inducing were harvested for further analyses. Quantitative real-time PCR revealed that the ε-, γ-, β-, ζ-, and α-globin genes were up-regulated in both K562 pcDNA and K562 miR-218 cells (Figure 6A–E), which demonstrated that the erythroid differentiation induced was successful. However, rising globin genes’ expression were repressed by ectopic expression of miR-218 compared to the K562 control cells (Figure 6A–E). In addition, the two most critical erythroid transcription factors gene, *GATA-1* and *KLF1*, were found significantly down-regulated in K562 miR-218 cells (Figure 6F). These results indicate that overexpression of miR-218 inhibits induced erythroid differentiation in K562 cells.

### 2.6. Hsa-miR-218 Plays a Role in the Proliferation and Apoptosis of K562 Cells

**Figure 7:**
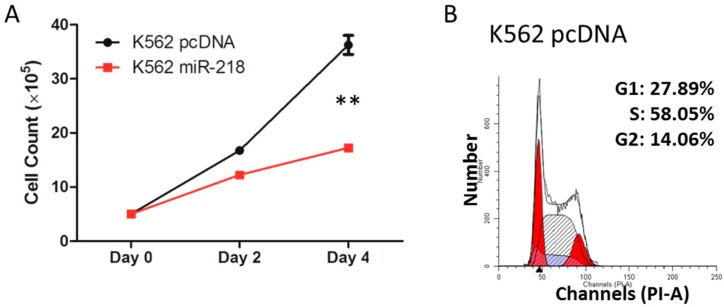

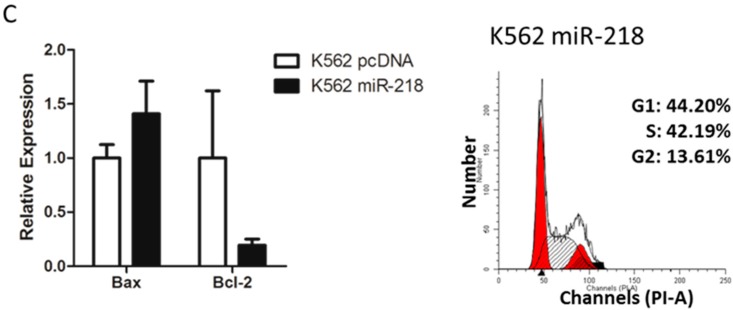
(**A**) Growth curve of control and K562 miR-218 cells. Cells were counted every two days. Asterisks indicate that differences between samples were statistically significant according to an independent-sample *t*-test, ** *p* < 0.01; (**B**) Cell-cycle assay of control and K562 miR-218 cells. Statistical percentages of each stage are shown; (**C**) Relative mRNA expression of *BAX* and *Bcl-2* in control K562 cells and K562 cells overexpressing miR-218 using quantitative real-time PCR normalized to *GAPDH*.

During the maintenance of the cell lines, a slower growth of the K562 miR-218 cells was observed compared to control cells. To verify that, 5 × 10^5^ control or K562 miR-218 cells were seeded respectively and cultured in same incubator, cells were harvest and counted every two days. The statistical results revealed that K562 miR-218 cells presented a relative slowly growth rate compared to control cells (Figure 7A). A cell cycle analysis (Figure 7B), indicated that more K562 miR-218 cells were blocked in the G1 stage than in the S stage. In addition, the expression of the pro-apoptosis gene BCL2-associated X protein (*BAX*) and anti-apoptosis gene B-cell CLL/lymphoma 2 (*BCL2* or *Bcl-2*) were quantified by quantitative real-time PCR (Figure 7C). *BAX* was up-regulated, while *Bcl-2* was down-regulated, due to miR-218 overexpression in K562 cells. These results indicate that miR-218 repressed the proliferation of K562 cells probably by cell cycle arrest and the induction of apoptosis.

## 3. Experimental Section

### 3.1. Cell Culture

The human erythroid leukemia cell line K562 was cultured in RPMI-1640 medium with 10% fetal bovine serum (FBS) at 37 °C in a 5% CO_2_ incubator. Hemin (Sigma, St. Louis, MO, USA) was used to induce K562 erythroid differentiation at a concentration of 0.05 mM.

### 3.2. Transcriptome, miRNome Sequencing, and Bioinformatics Analyses

RNA-seq and miRNA-seq were originally designed to explore the dynamic transcriptome and miRNome of human erythroid differentiation and development [17,29]. miRNome [30] sequencing was conducted using a Genome Analyzer II (Illumina, San Diego, CA, USA) according to the manufacturer’s protocol with read lengths of 50 bp. Data analyses were performed using miRDeep2 software [31]. The cleaned-up reads were mapped to the hg19 genomes and then the previously known and novel miRNA were identified using modules from miRDeep2. The expression level of each miRNA was normalized into corresponding transcripts per million (TPM): actual miRNA count × 1,000,000/total count of clean reads. The miRNome data have been deposited in the Gene Expression Omnibus database (accession numbers: GSE65706).

Four databases, miRanda [32], MicroCosm [32], microRNA.org [33] and TargetScan [34,35], were searched to identify computationally predicted miRNAs that target *ALAS2*.

An *ALAS2* interaction network was generated using Ingenuity Pathways Analysis (IPA) software (http://www.ingenuity.com/). This software was used to analyze data from a variety of experimental platforms and to provide accurate biological insights into interactions between genes, proteins, chemicals, pathways, cellular phenotypes, and disease processes.

### 3.3. Plasmids Construction

A siRNA specific to *ALAS2* (5′-GGAAACTATGTCTTCAGTT-3′) was designed by BLOCK-iT™ RNAi Designer (http://rnaidesigner.lifetechnologies.com), synthesized and cloned into plasmid pRNAT-U6.1/Neo (GenScript) to give pRNAT-ALAS2-sh. Primers were designed using the Primer 3 [36] designing tool in order to amplify primary sequences of miRNAs from human genomic DNA. The plasmid pcDNA3.0 (a kind gift from Dr. Yungui Yang, Beijing Institute of Genomics, Beijing, China) was used to generate a plasmid overexpressing miRNAs (e.g., pcDNA miR-218). pGL3-Basic vector (kindly provided by Dr. George Stamatoyannopoulos, University of Washington, Seattle, DC, USA) was altered to give pGL3-luc-MCS and pGL3-Rluc for the dual-luciferase reporter assay (Figure S2). The wild-type 3′UTR of *ALAS2* was cloned into pGL3.0-luc-MCS downstream of the luciferase reporter gene (*luc)* to give plasmids pGL3-luc-ALAS3-3′UTR. For mutated *ALAS2* 3′UTR, primers were designed by QuickChange Primer Design (Agilent Technology, Santa Clara, CA, USA). The QuickChange Lightning Site-Directed Mutagenesis Kit (Agilent Technology) was used to generate pGL3-luc-ALAS2-3′UTR-Mut. The sequences of the primers used are listed in Table S2 and Table S3. All of the constructs generated were confirmed by sequencing.

### 3.4. Total RNA Extraction and Quantitative Real-Time PCR

Total RNA was isolated from cells using TRIzol (Life Technology, Carlsbad, CA, USA) according to the manufacturer’s instructions. Complementary DNA (cDNA) was synthesized from 1 μg of total RNA using a First Stand synthesis Kit (Thermo Scientific, Waltham, MA, USA). Quantitative real-time PCR was performed on a BioRad CFX using SYBR Green/Rox chemistry (Thermo Scientific) according to the manufacturer’s instructions. The sequences of the primers used can be found in Table S4.

Expression of mature miRNA was determined by an All-in-One™ miRNA qRT-PCR Reagent Kit (GeneCopoeia, Rockville, MD, USA) according to the manufacturer’s instructions. Real-time PCR was then performed on a BioRad CFX.

### 3.5. Dual-Luciferase Reporter Assay

HEK293 or K562 cells were transiently transfected with pcDNA 3.0 or pcDNA miRNA, the firefly luciferase vector (pGL3-luc-ALAS3-3′UTR or pGL3-luc-ALAS2-3′UTR-Mut) and the Renilla luciferase vector (pGL3-Rluc). Forty-eight hours after transfection, cells were harvested and tested in a dual-luciferase reporter assay as previously described [37] in order to estimate the binding capacity of miRNA to the 3′UTR of *ALAS2*.

### 3.6. Mitochondria Isolation, Iron and Hemoglobin Quantification

Cells were harvested and lysed using RIPA cell lysis buffer (50mM Tris (pH 7.4), 150 mM NaCl, 1% NP-40 and 0.5% sodium deoxycholate). Mitochondria were isolated from the cells using a Qproteome Mitochondria Isolation kit (Qiagen, Germantown, MD, USA) according to the manufacturer’s instructions. The concentration of Fe^2+^ in the mitochondria was assayed using a QuantiChrom™ Iron Assay Kit (BioAssay Systems, Hayward, CA, USA) according to the manufacturer’s instructions. Readings were taken at OD_570nm_. Each experiment was performed in triplicate.

To evaluate the concentration of hemoglobin, cells were harvested and lysed in RIPA as described above. Total protein was quantified using the QuantiChrom™ Hemoglobin Assay Kit (BioAssay Systems) according to the manufacturer’s instructions. Each experiment was performed in triplicate.

### 3.7. Western Blot

Total protein was isolated from harvested cells using RIPA cells lysis buffer, and quantified using the BCA^TM^ Protein Assay Kit (Thermo Scientific, Rockford, IL, USA). Anti-*GAPDH* (ab75834) and anti-*ALAS2* (ab90574) antibodies were purchased from Abcam (Cambridge, MA, USA) and the secondary antibodies (e.g., anti-mouse and anti-rabbit) were purchased from ZSGB-BIO (Beijing, China). The band intensity was quantified by using ImageJ (National Institutes of Health, Bethesda, MD, USA).

### 3.8. Flow Cytometry

For cell surface CD235 analysis, 5 × 10^5^ cells were harvested and washed twice with phosphate buffered saline (PBS) and then re-suspended in 100 μL of PBS. The cells were incubated on ice in the dark for 10 min after adding 0.1 μL PE conjugated anti-CD235a (BD Biosciences, Bedford, MA, USA). The cells were washed twice by PBS, re-suspended in 500 μL of PBS, and then analyzed on a FACSCalibur flow cytometry (BD Biosciences).

The cell-cycle analysis was performed as previously described [38]. Cells were collected and washed twice by PBS, re-suspended in 1 mL of 75% (*v*/*v*) ethanol and fixed at −20 °C overnight. The ethanol was removed by centrifugation and the cells were suspended in PBS containing 10 μg/mL of RNase A for 30 min at 37 °C. Then the cells were stained with 50 μg/mL of propidium iodide (PI). DNA fluorescence was measured on a FACSCalibur (BD Biosciences) and analyzed with Modfit Lt V3.0 software (Verity Software House, Topsham, ME, USA).

### 3.9. Statistical Analysis

Data analysis was performed using GraphPad Prism® version 5.0 (GraphPad Software, Inc., La Jolla, CA, USA). Group data were expressed as mean ± SEM. Data were analyzed by standard statistical methods using an independent-sample Student’s *t*-test between two groups. *p* < 0.05 was considered to be significant.

## 4. Conclusions

This study aimed at exploring the role that specific miRNA(s) play in erythroid iron metabolism and differentiation. Both this and previous studies confirmed the role of *ALAS2* both in iron metabolism and erythroid differentiation. By using miRNA-seq and mRNA-seq data together with computational target prediction, seven candidate miRNAs that could potentially target *ALAS2* and thus may play a role in iron metabolism and erythroid differentiation were identified. Further experimental studies confirmed that *ALAS2* is a target for miR-218. Functional studies on miR-218 revealed that overexpression of miR-218 in K562 cells resulted in iron accumulation in mitochondria and inhibited erythroid differentiation. In all, our study found that miR-218 regulated iron metabolism and erythroid differentiation by targeting *ALAS2* in K562 cells.

## Supplementary Materials

Supplementary materials can be found at http://www.mdpi.com/1422-0067/16/12/26088/s1.
