# Supplementary material for: miR-218 Inhibits Erythroid Differentiation and Alters Iron Metabolism by Targeting ALAS2 in K562 Cells
Source: Int J Mol Sci. 2015 Nov 26;16(12):28156–68. doi: 10.3390/ijms161226088 (PMC4691035; doi:10.3390/ijms161226088)
Supplement: Supplementary file 1 [file ijms-16-26088-s001.pdf]

Supplemental Materials: miR-218 Inhibits Erythroid Differentiation and Alters Iron Metabolism by Targeting *ALAS2* in K562 Cells

Yanming Li, Shuge Liu, Hongying Sun, Yadong Yang, Heyuan Qi, Nan Ding, Jiawen Zheng, Xunong Dong, Hongzhu Qu, Zhaojun Zhang and Xiangdong Fang

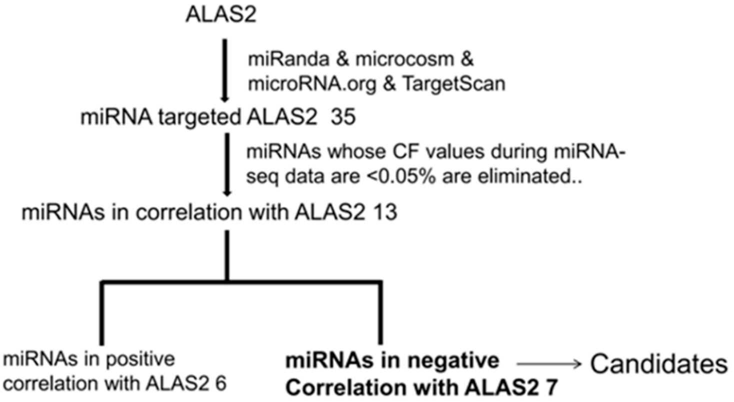

Figure S1. Methodology of screening candidate miRNAs.

Table S1. Expression (CF) pattern of miRNAs which may targeting *ALAS2* with inverse correlation with *ALAS2*.

miRNA	HESC	ESER	FLER	PBER
hsa-miR-124	75.226	0.147	3.074×10^{-4}	0.032
hsa-miR-206	0.500	9.198×10^{-4}	2.043×10^{-6}	0
hsa-miR-218	0.051	1.004×10^{-4}	2.101×10^{-7}	0
hsa-miR-222	119.028	0.232	4.864×10^{-4}	4.200
hsa-miR-330-3p	13.583	0.026	5.551×10^{-5}	5.818
hsa-miR-342-5p	0.288	5.627×10^{-4}	1.178×10^{-6}	0.243
hsa-miR-518d-5p	0.081	1.579×10^{-4}	3.310×10^{-7}	0

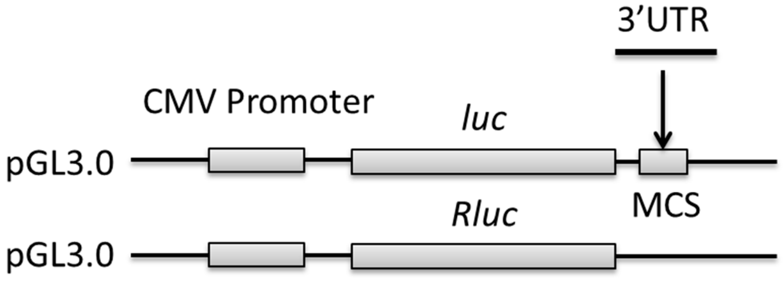

Figure S2. Vectors applied in dual-luciferase reporter assay.

Table S2. Primers sequences for ampification of primiary miRNAs.

Traget	Primer Sequence (5′–3′)
Pri-miR-124	GGGCTCGAGCTGTAAATGGCATGGAGATATA<br>GGGGCGGCCGCGGAGATTTGCCTGCGGATTT
Pri-miR-206	GGGCTCGAGCTGCCATTCCTCACAACAGA<br>GGGGCGGCCGCTGAGGGAGTCATTTGCTG
Pri-miR-218	GGG CTCGAGCAGTTCTGAGGAATGGTGGA<br>GGG GCGGCCGCTCTAGCCTACTTCACAGCTA
Pri-miR-222	GGGCTCGAGAGCCCCAGCTGATAATGTTGGACT<br>GGGGCGGCCGCATGTCACTCAGTCAGTATCTGTTGG
Pri-miR-330	GGGCTCGAGCCACTCACCCACACTGAAGA<br>GGGGCGGCCGCGTTTCTCCCTCTGCTTGACG
Pri-miR-342	GGGCTCGAGGGGCAGGTGTCTGTTTCTGT<br>GGGGCGGCCGCTCACAGATGGCGAAACTGAG
Pri-miR-518	GGGCTCGAGAAAACAGGCAAACAGGACCA<br>GGGGCGGCCGCGCACAGAACCCCGATATCAT

Table S3. Primers for ampification of 3′UTR or mutated 3′UTR.

Traget	Primer Sequence (5′–3′)
VIM-3′UTR	GGGGCTAGCCAGCATCACGATGACCTT<br>GGGCTCGAGGAAGTGAGGTCTATCAAAAATG
ALAS2-3′UTR	GGGCTCGAGCATGCTGTGGTTTGTGCTTT<br>GGGGCTAGCGTATGTCACCACCTATGCCT
ALAS2-3′UTR-M1	GTGCCTCTAGCTGAATTGAGCCTAAAAATACTCCACAAACCACAGCATG<br>CATGCTGTGGTTTGTGGAGTATTTTTAGGCTCAATTCAGCTAGAGGCAC
ALAS2-3′UTR-M2	CCTCTAGCTGAATTGAGCCTAAAAATAAAGCAGCCACCACAGCATGTGAAGC<br>GCTTCACATGCTGTGGTGGCTGCTTTATTTTTAGGCTCAATTCAGCTAGAGG
ALAS2-3′UTR-M4	TCTTCTGCTTTGTTGTGTGCCTCTAGCTGAATTGAGCCTA<br>AAAATAGGATGTGAACCACAGCATGTGAAGCCTTTTA<br>TAAAAGGCTTCACATGCTGTGGTTCACATCCTATTTTTAG<br>GCTCAATTCAGCTAGAGGCACACAACAAAGCAGAAGA

Table S4. Primer sequences of quantitative real-time PCR.

Traget	Primer Sequence (5′–3′)
GAPDH	TGTTGCCATCAATGACCCCTT<br>CTCCACGACGTACTCAGCG
β-actin	ATAGCACAGCCTGGATAGCAACGTAC<br>CACCTTCTACAATGAGCTGCGTGTG
ALAS2	CTACCCAAGGACCAAACTGTTC<br>TGCCTTCTGCACAATCTTGCT
IRP2	TCGATGTATCTAAACTTGGCACC<br>GCCATCACAATTTCGTACAGCAG
Ferritin	CAGCCTGGTCAATTTGTACCT<br>GCCAATTCGCGGAAGAAGTG
TFR1	ACCATTGTCATATACCCGGTTCA<br>CAATAGCCCAAGTAGCCAATCAT
Transferrin	GGTGGCAGAGTTCTATGGGTC<br>ACAGTAAAGTAAGCCTATGGGGA
HBA1	CAGGAACTTGTCCAGGGAGG<br>GGACCCGGTCAACTTCAA
HBZ	AGTGCGGGAAGTAGGTCTTG<br>TGACCAAGACTGAGAGGACCA
HBE	GGTGCATTTTACTGCTGAGGA<br>CCCATGCATTGAGAACCAA
HBG	GCCATGGGTCATTTCACAG<br>TGGTCACCAGAGCCTACCTT
HBB	ATCAGAAAGTGGTGGCTGGT<br>GATGCTCAAGGCCCTTCATA
GATA-1	CCAGACGACCACCACGACA<br>TCCTTCGGCTGCTCCTGTG
KLF1	TTGCGGCAAGAGCTACACC<br>GTCAGAGCGCGAAAAAGCAC
